# Cu Mesh for Flexible Transparent Conductive Electrodes

**DOI:** 10.1038/srep10715

**Published:** 2015-06-03

**Authors:** Won-Kyung Kim, Seunghun Lee, Duck Hee Lee, In Hee Park, Jong Seong Bae, Tae Woo Lee, Ji-Young Kim, Ji Hun Park, Yong Chan Cho, Chae Ryong Cho, Se-Young Jeong

**Affiliations:** 1 Department of Cogno-Mechatronics Engineering, Pusan National University, Miryang, 627-706 (Republic of Korea); 2 Department of Materials Science and Engineering, University of Maryland, College Park, MD 20742 (United States); 3 Department of Nanofusion Engineering, Pusan National University, Busan, 609-735 (Republic of Korea); 4 Busan Center, Korea Basic Science Institute, Busan, 618-230 (Republic of Korea); 5 KAIST Analysis Center for Research Advancement, Daejeon, 305-701 (Republic of Korea); 6 Division of General Studies, Ulsan National Institute of Science and Technology (UNIST), Ulsan, 689-798 (Republic of Korea); 7 Frontier in Extreme Physics, Korea Research Institute of Standards and Science, Daejeon, 305-340, (Republic of Korea)

## Abstract

Copper electrodes with a micromesh/nanomesh structure were fabricated on a polyimide substrate using UV lithography and wet etching to produce flexible transparent conducting electrodes (TCEs). Well-defined mesh electrodes were realized through the use of high-quality Cu thin films. The films were fabricated using radio-frequency (RF) sputtering with a single-crystal Cu target—a simple but innovative approach that overcame the low oxidation resistance of ordinary Cu. Hybrid Cu mesh electrodes were fabricated by adding a capping layer of either ZnO or Al-doped ZnO. The sheet resistance and the transmittance of the electrode with an Al-doped ZnO capping layer were 6.197 ohm/sq and 90.657%, respectively, and the figure of merit was 60.502 × 10^–3^/ohm, which remained relatively unchanged after thermal annealing at 200 °C and 1,000 cycles of bending. This fabrication technique enables the mass production of large-area flexible TCEs, and the stability and high performance of Cu mesh hybrid electrodes in harsh environments suggests they have strong potential for application in smart displays and solar cells.

Transparent conducting electrodes (TCEs), which have high optical transparency and high electrical conductivity, are widely used in numerous optoelectronic devices, including organic/inorganic light-emitting diodes, liquid-crystal displays, touch-screen panels, supercapacitors, and solar cells^1^,^2^,^3^,^4^,^5^,^6^,^7^. Historically, the use of indium tin oxide (ITO) has dominated in the TCE industry; however, some critical problems are associated with the mechanical, thermal and chemical stability of ITO. The scarcity of indium and its increasing price are additional factors that make ITO undesirable in TCE applications. Thus, efforts have been devoted to the development of alternatives to ITO (e.g., conducting polymers, Al-doped ZnO and metal thin films); however, these alternatives have been unsatisfactory for industrial demands^8^,^9^,^10^. In recent years, however, micromaterials and nanomaterials, including carbon nanotubes, graphene, and metal wire/particle networks, have offered a new paradigm for the TCE industry^11^,^12^,^13^,^14^,^15^,^16^,^17^,^18^,^19^,^20^. Among these, metal nanostructure network TCEs have exhibited high performance and are suitable for large-area applications. Various fabrication methods have been employed, such as spin (or spray) coatings of nanowire dispersions^11^,^12^,^13^,^14^,^15^,^16^,^17^, electronic spinning^17^,^18^,^19^, and lift-off processes with nanotemplates^21^,^22^,^23^. Silver (Ag), in particular, has attracted great interest for TCE applications because of its superior electrical conductivity, and various fabrication processes have been investigated to simplify the manufacturing process and to improve performance^4^,^13^,^14^,^15^,^16^,^17^,^18^,^20^,^21^,^22^. However, Ag is not suitable for use in mass production requiring low costs. Copper (Cu) has high electrical conductivity comparable to that of Ag, and Cu is much less expensive and more abundant than Ag^11^,^23^. Recently, transparent conductive films have been reported that are formed of Cu nanowires and exhibit good electrical and mechanical performance. These films have potential applications in low-cost and mass—produced electronic devices^11^,^23^.

In this study, we demonstrate a method of fabricating mesoscale Cu mesh structures for TCE applications. To fabricate a high-quality Cu thin film on a polyimide substrate, we employed a conventional RF sputtering method with a single-crystal Cu target, thereby improving the oxidation resistance and the adhesive force of the Cu thin film^24^. All microscale/nanoscale mesh structures were produced using only conventional UV lithography and wet etching, thereby avoiding complicated and expensive methods such as e-beam lithography or nanoimprint lithography^3^,^19^. We examined the electrical conductivity, the mechanical stability and the oxidation resistance of the Cu mesh to assess its practical applicability. In addition, we fabricated several types of capping layers on the Cu mesh structures to enhance their electrical characteristics and their chemical stability without degrading their transmittance.

The structure of the Cu mesh hybrid electrode is illustrated in Fig. 1(a), where the Cu mesh layer was given a honeycomb structure, which resulted in the greatest mechanical stability and transmittance among several structures we tested. Cu thin films of ~60 nm were prepared on polyimide (PI) substrates using RF sputtering using either a single-crystal Cu target or a polycrystalline Cu target with approximately equal purities (99.995% and 99.999%, respectively) for comparison (the single-crystal Cu target was supplied by the Crystal Bank at Pusan National University). The Cu thin films fabricated using the polycrystalline Cu target and the single-crystal Cu target are abbreviated as PCu and SCu, respectively. The physical characteristics of the Cu thin films fabricated using the two types of targets have been reported in detail in a previous study^24^. Although the PCu and SCu thin films were deposited on an amorphous polymer substrate, both films were oriented along the (111) direction. Figure 1(b) and (c) show atomic force microscopy (AFM) images of the PCu and SCu thin films, respectively. The PCu thin film has a random distribution of grains of various sizes from 20 nm to 200 nm, whereas the SCu film has a homogenous distribution of grains from 20 nm to 40 nm in size. In the previous study^24^, the sputtering process used the homogeneous interatomic bonding energy of a grain-free single-crystal Cu target to enable the fabrication of high-quality Cu thin films (111) on Al_2_O_3_ (0001) substrates, which are nearly perfect single crystals, with a highly oriented Bragg plane along the (111) direction. Figure 1(b) and (c) show evidence that a Cu thin film deposited on an amorphous substrate such as polyimide also strongly depends upon the type of Cu target and that the quality of the film can be enhanced considerably by employing a single-crystal Cu target during the sputtering process.

The diameters of the hexagonal open areas on the honeycomb-patterned photoresist mask were varied from 5 to 30 μm. The line width of the honeycomb pattern on the photoresist mask was fixed at 3 μm, and the line width of the honeycomb pattern on the Cu mesh electrode was reduced to hundreds of nanometers by wet etching with various processing times. The difference between the PCu and SCu mesh electrodes was noticeable after the wet etching process. Figure 1(d) and (e) show the AFM images of the PCu and SCu honeycomb mesh electrodes fabricated using UV lithography and wet etching. The wet etching process is strongly affected not only by the crystallinity, the crystal direction and defects, but also by the grain size and the grain size distribution^25^. Etching preferentially occurred around the inhomogeneous grains and the crystal defects in the PCu mesh electrode; thus the conduction path of the honeycomb mesh was disconnected in many places (see Fig. 1(d)), which precluded the possibility of creating a PCu mesh with a line width less than 1 μm using the undercut process^22^. In contrast, the SCu mesh electrode exhibited a well-defined honeycomb structure, as shown in Fig. 1(e), and the line width of the honeycomb pattern could be etched down to hundreds of nanometers because the homogeneous, small grains of the SCu thin film allowed a consistent etching process over the entire SCu mesh electrode. The etching rates were nearly the same on the SCu mesh electrode regardless of the diameter of the hexagons; however, the line widths were manipulated through variation of the time of the wet etching process (see Fig. S1 in Supplementary Information). However, at line widths less than approximately 200 nm, the conductor paths tended to collapse and the sheet resistance drastically increased (see Fig. S2 in Supplementary Information). Therefore, we deduced that the line width limit of the honeycomb mesh is approximately 200–300 nm and that the application of an additional passivation process is necessary to fabricate line widths less than 200 nm using wet etching^26^. The SCu layer was quite stable against oxidation; however, for better protection, capping layers of either a ZnO or Al-doped ZnO (AZO) layer were fabricated on the SCu mesh electrodes using RF sputtering (Fig. 1(a) and (f)). Figure 1(f) shows an illustration and high-resolution transmission electron microscopy (HRTEM) image of the cross-sectional structure of a Cu mesh hybrid electrode with an AZO capping layer and a polyimide substrate. The HRTEM image was obtained from the AZO-capped Cu mesh electrode formed using a single-crystal Cu target (i.e., Fig. 1(e)), which shows a structure that is consistent with the design (see the illustration).

In Fig. 2(a), we show a plot of the optical transmittance (*T*) of the SCu mesh electrodes as a function of the sheet resistance (*R*_*s*_) to identify the optimum line width and hexagon size (2r_i_). We fabricated the SCu mesh electrodes with line widths of 3 μm, 1 μm, 500 nm and 350 nm and with hexagon sizes of 5, 10, 15, 20, 25 and 30 μm, which are abbreviated as D5, D10, D15, D20, D25 and D30, respectively. The transmittance *T* tended to increase as the covered area decreased when the line width was decreased from 3 μm to 350 nm; however, *R*_*s*_ also increased because of the narrowing of the conduction path. Moreover, both *T* and *R*_*s*_ increased in proportion to the reduction of the covered region per unit area with the increase in the hexagon size from 5 μm to 30 μm. The mesh with a hexagon size D5 and a line width of 3 μm exhibited the best sheet resistance (1.085 ohm/sq) and the worst transmittance (29.589%), whereas the mesh with a hexagon size D30 and a line width of 350 nm exhibited the worst sheet resistance (41.310 ohm/sq) and the best transmittance (97.146%). At the μm scale, the distribution of the sheet resistance as a function of D was narrower than at the nm scale. This is because the variation in the fraction of the etched areas as a function of the line width was smaller for a thicker line width. Note that the sheet resistance of SCu (1mm:D15) was similar to that of SCu (1mm:D20), which appears to be determined primarily by the control over the line width. To determine the optimum line width and hexagon size, we calculated the figure of merit (FoM) as defined by Haacke^27^:
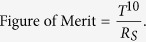


Figure 2(b) and (c) show the FoM as a function of the line width and the hexagon size, and a photograph of hybrid electrode AZO/SCu (1 μm:D30), demonstrating optical high transparency, respectively. The maximum FoM, 46.47 × 10^−3^/ohm, was achieved with the D30 hexagon size and with a line width of 1 μm, i.e., (1 μm:D30), where *R*_*s*_ and *T* were 10.372 ohm/sq and 92.962%, respectively. To enhance the FoM, we deposited capping layers of either ZnO or AZO with a thickness of 75 nm onto SCu(1 μm:D30) meshes, which had the best FoM, using RF sputtering; these electrodes are abbreviated as ZnO/SCu(1 μm:D30) and AZO/SCu(1 μm:D30). These hybrid electrodes, ZnO/SCu(1 μm:D30) and AZO/SCu(1 μm:D30), retained high transparency—greater than 90%—but their optical transmittances of 90.908% and 90.657%, respectively were slightly less than that of SCu (1 μm:D30) because of the ZnO (T_550nm_ = 92.204%) or AZO (T_550nm_ = 91.837%) capping layer. However, the *R*_*s*_ improved slightly, decreasing to 7.747 ohm/sq for ZnO/SCu(1 μm:D30) and 6.197 ohm/sq for AZO/SCu (1 μm:D30).

Although both ZnO (*R*_*s*_ = 188,260 ohm/sq) and AZO (*R*_*s*_ = 7,854 ohm/sq) alone have much higher resistance than the SCu mesh electrode, when either is layered on top of the SCu mesh, the resistance improves by an order of magnitude, which can be explained by the percolation effect. The overlaid ZnO and AZO form a co-percolation network as an additional pathway for charge transfer by filling the hexagons in the SCu mesh^28^,^29^,^30^. The FOMs of ZnO/SCu(1 μm:D30) and AZO/SCu(1 μm:D30) exhibited significant improvements, 46.762 × 10^−3^/ohm and 60.502 × 10^−3^/ohm, respectively, because of a decrease in *T* of 2.210% and 2.480%, respectively, and a decrease in *R*_*s*_ of 25.312% and 40.248%, respectively.

Recently TCEs require thermal stability at high temperature for flexible device applications in harsh environments in addition to resistance to oxidation. The most serious problem with Cu electrodes is their low oxidation resistance. Oxidation becomes more important in microscale/nanoscale devices such as mesh structures because of the abrupt extension of the surface area. We investigated the changes in *R*_*s*_ in the four types of electrodes—PCu mesh, plain SCu mesh and SCu mesh with a capping layer of ZnO or AZO—resulting from thermal annealing at various temperatures. To determine the oxidation resistance of each sample, the resistance was measured under the same environmental conditions. The changes in resistance *R*_*s(T)*_/*R*_*s(0)*_, where *R*_*s(T)*_ is the sheet resistance at temperature *T* and *R*_*s(0)*_ is the initial sheet resistance before thermal annealing, were plotted as a function of temperature. Figure 3(a) shows that the electrical properties of the PCu electrode with a line width of 3 μm and a hexagon size of 30 μm (PCu(3 μm:D30)) and those of the plain and hybrid SCu electrodes with a line width of 1 μm and a hexagon size of 30 μm (SCu(1 μm:D30), ZnO/SCu(1 μm:D30), and AZO/SCu(1 μm:D30)) all strongly depend on the annealing temperature.

The normalized sheet resistance for PCu(3 μm:D30) increased gradually at lower annealing temperatures, in approximate proportion to the annealing temperature. An abrupt increase due to oxidation was observed near 150 °C, and the resistance exceeded the measurable limit above 150 °C. SCu(1 μm:D30) exhibited a smaller increase in *R*_*s(T)*_/*R*_*s(0)*_ and greater oxidation resistance than PCu(3 μm:D30). According to the results of previous studies^24^ and those presented in Fig. 1(b)–(e), oxidation is less likely to occur below the surface in SCu electrodes because single-crystal copper and the SCu thin film exhibit high crystallinity, a homogeneous grain distribution, and few defects. However, the SCu electrode developed significant oxidation above 175 °C. The capping layer was introduced to limit oxidation and proved to be very effective. The normalized sheet resistance for both ZnO/SCu(1 μm:D30) and AZO/SCu(1 μm:D30) remained well within the measurable range, even at 200 °C, where the values were only 1.52 and 1.2 times greater, respectively, than those at room temperature.

To investigate the penetration of oxidation into the electrodes, we used X-ray photoelectron spectroscopy (XPS) to measure the Cu 2*p*_3/2_ depth profile before and after the electrodes were thermally annealed at 200 °C, as shown in Fig. 3(b). In the case of PCu(3 μm:D30), the Cu_2_O peak (932.6 eV) before the electrode was annealed, marked with asterisk, was observed in the spectrum collected at the surface; however, after the sample was thermally annealed at approximately 200 °C, the Cu_2_O peak was detected in the spectrum measured inside the Cu layer. For SCu(1 μm:D30), the Cu_2_O peak was initially observed in the spectrum measured at the surface; however, the penetration of the oxidation into the Cu layer was noticeably smaller compared with that of PCu(3 μm:D30) after the electrodes were thermally annealed at approximately 200 °C. Notably, the capping layers—ZnO and AZO—stabilized the SCu layer against thermal annealing, where the Cu 2*p*_3/2_ profiles of both samples showed no shift in the peaks associated with the Cu layer after the electrodes were thermal annealed at approximately 200 °C. A broad peak at approximately 935.1 eV, marked by a cross (†), corresponded to Cu(OH)_2_, and this peak clearly disappeared after H_2_O evaporated during the thermal annealing. The XPS depth profile of O 1*s* in the Cu layer provides further evidence of the degree of oxidation of the Cu (see Fig. 3(c)). In the case of PCu(3 μm:D30), the O peak (531.5 eV) was initially at the surface; however, after the samples were thermally annealed at approximately 200 °C, the O peak appeared in the spectrum collected inside the Cu layer, as was observed with Cu 2*p*_3/2_. However, no O 1*s* peak for copper oxide was observed in SCu(1 μm:D30), except at the narrow interface region. In particular, the spectra of the samples with a capping layer, ZnO/SCu(1 μm:D30) and AZO/SCu(1 μm:D30), contained no peak related to oxygen, even at the interface regions. However, the spectra of the surfaces of the capping layers of ZnO and AZO showed slightly increased peak intensities for carbonates (531.0 eV) and hydroxides (531.5 eV) in addition to the peak for ZnO (530.0 eV) because of oxidation, as shown in Fig. S3 (see Supplementary Information). This oxidation may have caused the increase of *R*_*s*_ in the SCu mesh hybrid electrodes by reducing the free carriers in the capping layer due to the curing of the oxygen vacancies (V_o_) in ZnO rather than by the oxidation of SCu. In addition, the greater decrease in *R*_*s*_ in AZO/SCu(1 μm:D30) than in ZnO/SCu(1 μm:D30) (Fig. 3(a)) is attributed to the Al dopant in the AZO generating not only V_o_ but also free carriers so that the electrical properties are preserved when the oxygen vacancies are cured^31^. Consequently, we conclude that AZO is a better capping-layer material than ZnO because the former not only has a higher a FoM for a hybrid TCE but also protects the SCu mesh layer from oxidation and retains the low sheet resistance.

More recent requirements for TCEs include mechanical stability in addition to the thermal stability. To determine which type of TCE would perform best under external mechanical stress, we measured the change in resistance after repeated bending cycles. The samples were flexed to a radius of curvature of 2 mm (see inset of Fig. 4(a)), and the resistance was measured after a certain number of bending cycles. This process of flexing and measuring the resistance was continued until 1000 cycles were completed. The change in resistance is represented by *R*_*s(n)*_/*R*_*s(0)*_ in Fig. 4(b), where *R*_*s(n)*_ is the sheet resistance after *n* bending cycles.

As expected, the results for PCu(3 μm:D30) and SCu(1 μm:D30) were noticeably different, as is evident in Fig. 4(b). The resistance of PCu(3 μm:D30) drastically increased with increasing number of bending cycles; however, the resistance of SCu(1 μm:D30) remained nearly unchanged after 1000 bending cycles, even though SCu(1 μm:D30) had a smaller line width than PCu(3 μm:D30) (Fig. 4(b)). With or without a capping layer, all of the SCu mesh samples exhibited high mechanical stability, with a change in resistance of less than 8% after 1000 bending cycles. The reason for the abrupt increase in resistance in the PCu mesh electrode is that the wet etching process exposes the mesh structure with inhomogeneous grains, and thus the external mechanical stress is not equally distributed, which leads to cracking and disconnection of the conduction paths. In contrast, the SCu mesh electrodes have a well-defined mesh structure because of the small and homogeneous grains and the low number of defects, resulting in high mechanical stability under bending. The presence of a capping layer of either ZnO or AZO had no effect on the mechanical stability. These optical, electrical, chemical, and mechanical characteristics show that the performance of the SCu mesh hybrid electrodes is comparable to that of existing technologies and could overcome the various obstacles of ordinary Cu.

## Conclusion

Copper is generally the best material for electrodes; however, the use of Cu in a transparent electrode faces several challenges. The Cu thin film deposition process cannot avoid oxidation; thus, the resistivity of the film is very large. Moreover, both high conductivity and high transparency cannot be achieved in a mesh structure fabricated from a Cu thin film. The Cu mesh structure exhibits poor adhesion to the substrate, especially a flexible substrate, which allows the mesh to easily separate from the substrate. These problems have prevented Cu mesh electrodes from being used in mass production and large-area applications.

We were able to overcome these problems in this study. Using RF sputtering with a single-crystal Cu target, we grew high-quality copper thin films with a high degree of homogeneity and crystallinity without resorting to molecular beam epitaxy, pulsed laser deposition, and chemical vapor deposition. We were able to subsequently fabricate micromesh/nanomesh structures on a flexible substrate using only UV lithography and wet etching instead of costly e-beam and nanoimprint processes.

The FoM was used to determine the mesh with the most effective line width and mesh size; the best results were obtained from a honeycomb mesh with a 1 μm line width and a 30 μm hexagon size. After the deposition of an AZO capping layer, which created a co-percolation effect, the transparency, the surface resistance and the FoM were 90.657%, 6.197 ohm/sq, and 60.502 × 10^−3^/ohm, respectively, and the Cu mesh was effectively protected from oxidation. This hybrid mesh electrode maintained high mechanical and electrical stability after 1,000 cycles of repeated bending stresses.

We believe that the high performance of the plain and hybrid SCu mesh electrodes makes them suitable for applications in various flexible optoelectronic devices and that they could be used in other mechanical, medical, electronics and energy applications.

## Methods

### Cu thin film

Cu thin films with a thickness of 62 nm were deposited on a polyimide substrate (Neopulim L-3430, Mitsubishi Gas Chemical Co., Inc.) at 150 °C in an atmosphere of Ar (99.999%) gas using RF sputtering. A high-purity polycrystalline Cu target (99.999%) and a single-crystal Cu target (99.995%) were used for comparison. A single-crystal Cu ingot was obtained using the Czochralski method, and this ingot was prepared in the form of disk targets with a diameter of 2 inches using an electrical-discharge machining process.

### Cu mesoscale mesh transparent electrodes

A photoresist layer (AZ1512, AZ Electronic Materials) was spin-coated for 30 seconds onto Cu thin films rotating at 4000 rpm and were then soft-baked for 2 minutes at 105 °C on a hotplate. A honeycomb mesh structure was transferred onto the photoresist layer using UV lithography. The regions exposed to UV light on the photoresist layer were removed with a developer (MIF300, AZ Electronic Materials), and, after the sample was rinsed with distilled water, the patterned sample was hard-baked at 110 °C for 10 minutes.

The samples were wet-etched at 25 °C using a Cu etchant, which was prepared from acetic acid (CH_3_COOH), hydrogen peroxide (H_2_O_2_), and distilled water in a 1:1:10 (by volume) ratio. The undercut depth was controlled by adjusting the etching time, which was approximately 8.3 nm/s for the SCu thin films. Subsequent to the etching process, the photoresist masking layer was removed using sonication and acetone, and the samples were rinsed with ethanol and then distilled water.

### ZnO(:Al) capping layer

Capping layers were deposited on the SCu mesh films at 150 °C in an Ar (99.999%) gas atmosphere using RF sputtering, where pure polycrystalline ZnO and 2 mol% Al-doped ZnO were used as targets.

### Optical, electrical, and structural characterizations

The optical transmittance of the Cu mesh electrodes was measured using a UV-Vis spectrophotometer (Cary 5000, Varian); the baseline correction procedure was executed with a polyimide substrate. The sheet resistance of the Cu mesh electrodes was measured using a Hall-effect measurement system (HMS-3000, Ecopia) with the van der Pauw technique. The surface morphology of the Cu mesh electrodes was obtained using an atomic force microscope (XE-100, Park systems) equipped with a noncontact cantilever (PPP-NCHR, Nanosensors); cross-sectional images of the bilayer structure were obtained using a transmission electron microscope (Tecnai TF300 ST, FEI Company).

### Thermal and mechanical stability measurements

We evaluated the thermal stability and the oxidation resistance of the Cu mesh electrodes by measuring the change in the sheet resistance after the electrodes were thermally annealed in air. Each measurement was taken at room temperature after the electrodes were annealed at temperatures from 50 °C to 200 °C in 25 °C increments using the rapid thermal annealing method. The shifts in the chemical binding energies of Cu, O, and Zn were measured using an X-ray photoelectron spectrometer (Korea Basic Science Institute, Busan Center). The mechanical stability of the Cu mesh electrodes was investigated by measuring the change in the sheet resistance as a function of the number of bending cycles, where the Cu mesh electrodes were flexed manually to a radius of curvature of 2 mm.

## Additional Information

**How to cite this article**: Kim, W.-K. *et al.* Cu Mesh for Flexible Transparent Conductive Electrodes. *Sci. Rep.* **5**, 10715; doi: 10.1038/srep10715 (2015).

## Supplementary Material

Supplementary Information

## Figures and Tables

**Figure 1:**
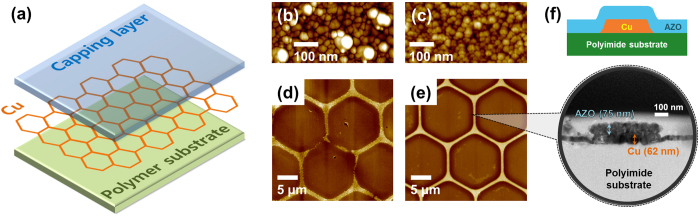
(**a**) Structure of the Cu mesh hybrid electrode. (**b**) and (**c**) AFM topography images of Cu thin films on polyimide substrates fabricated using a polycrystalline Cu target and a single-crystal Cu target, respectively. (**d**) and (**e**) AFM topography images of Cu mesh electrodes fabricated with wet etching using the two types of Cu thin films in (**b**) and (**c**), respectively. (**f**) Illustration and HRTEM image of the cross-sectional structure of a Cu mesh hybrid electrode with Al-doped ZnO as the capping layer and polyimide as the polymer substrate.

**Figure 2:**
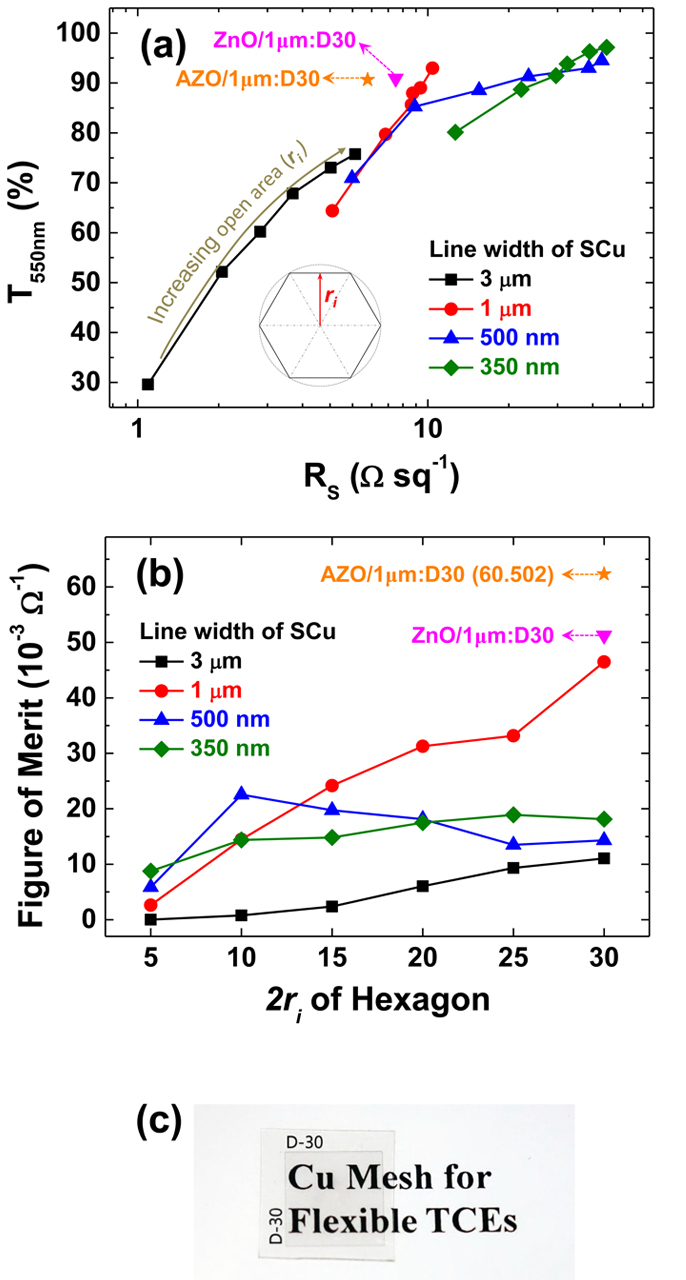
(**a**) Sheet resistance vs. transmittance (R_s_-T) for SCu mesh electrodes with various hexagon diameters and line widths: ■ - 3 μm, ● - 1 μm, ▲ - 500 nm, and ♦ - 350 nm, ▼ - ZnO capping layer/SCu mesh hybrid electrode, and ★ - Al-doped ZnO capping layer/SCu mesh hybrid electrode. ZnO/SCu(1 μm:D30) and AZO/SCu(1 μm:D30) are abbreviations for an SCu mesh with a line width of 1 μm, a distance between opposite sides of hexagon (2r_i_) of 30 μm and a capping layer of ZnO and AZO, respectively. The transmittance and the surface resistivity tended to increase with decreasing line width and increasing diameter. (**b**) Figures of merit of SCu mesh electrodes and hybrid electrodes as a function of hexagon size. (**c**) Photograph of hybrid electrode AZO/SCu(1 μm:D30), which had the highest figure of merit.

**Figure 3:**
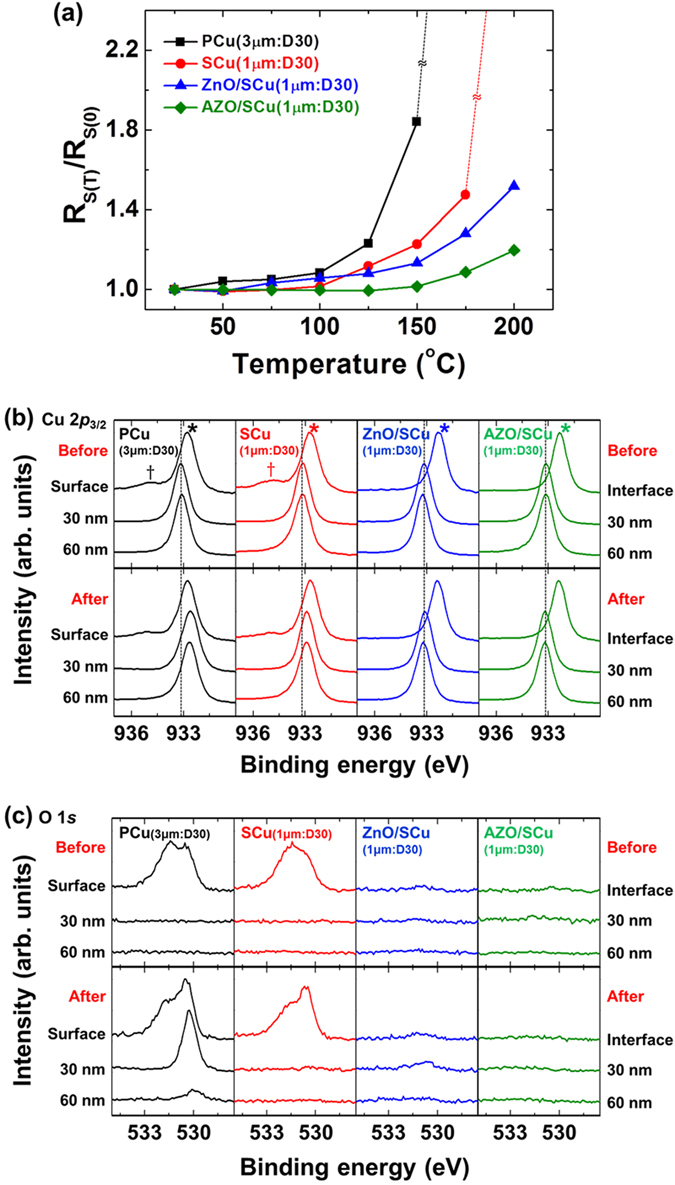
(**a**) Changes in the normalized sheet resistance of the PCu mesh electrode, the plain SCu mesh electrode and the SCu mesh hybrid electrodes after being thermally annealed in air. X-ray photoelectron spectroscopy (XPS) depth profiles of (**b**) Cu 2*p*_3/2_ and (**c**) O 1*s*, respectively, in the Cu films in the electrodes before and after the electrodes were thermally annealed at 200 °C. In (**b**), the asterisk (*) at 932.6 eV indicates Cu_2_O, and the cross (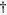) at the broad peak at approximately 935.1 eV indicates Cu(OH)_2_, which disappeared after the electrodes were annealed because of the evaporation of H_2_O. The depth profiles begin at the surface for the PCu and plain SCu meshes and at the interface between the capping layer and the Cu mesh for the hybrid electrodes.

**Figure 4:**
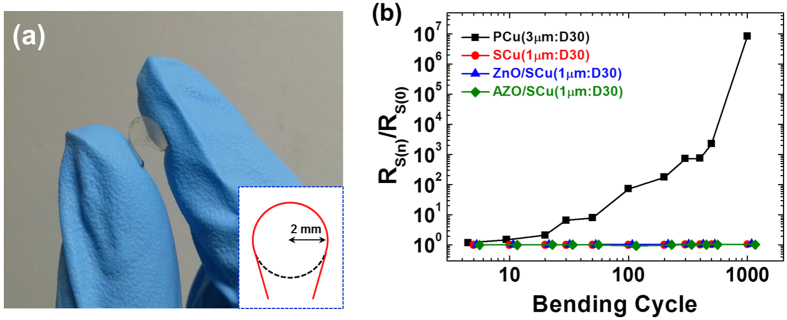
(**a**) Photograph of mechanical stability tests of flexible mesh electrodes. (Inset: drawing showing the diameter of the sample in the bending tests.) (**b**) Effect of repeated flexing on the normalized sheet resistance.
