# Supplementary material for: Cu Mesh for Flexible Transparent Conductive Electrodes
Source: Sci Rep. 2015 Jun 3;5:10715. doi: 10.1038/srep10715 (PMC4454070; doi:10.1038/srep10715)
Supplement: Supplementary Information [file srep10715-s1.doc]

**Cu Mesh for Flexible Transparent Conductive Electrodes**

*Won-Kyung Kim*^1^, *Seunghun Lee*^1[+]^, *Duck Hee Lee*^2^, *In Hee Park*^2^, *Jong Seong Bae*^3^, *Tae Woo Lee*^4^, *Ji-Young Kim*^1^, *Ji Hun Park*^1^, *Yong Chan Cho*^5^, *Chae Ryong Cho*^6^, *Se-Young Jeong*^1*^

^1^ Department of Cogno-Mechatronics Engineering, Pusan National University, Miryang, 627-706 (Republic of Korea)

* e-mail : [syjeong@pusan.ac.kr](mailto:syjeong@pusan.ac.kr)

^2^ Department of Nanofusion Engineering, Pusan National University, Busan, 609-735 (Republic of Korea)

^3^ Busan Center, Korea Basic Science Institute, Busan, 609-735 (Republic of Korea)

^4^ KAIST Analysis Center for Research Advancement, Daejeon, 305-701 (Republic of Korea)

^5^ Crystal Bank Institute, Pusan National University, Miryang, 627-706 (Republic of Korea)

^6^ Department of Nano Fusion Technology, Pusan National University, Miryang, 627-706 (Republic of Korea)

^[+]^ *Current address* : The Institute of Basic Science, Korea University, Seoul, 136-713 (Republic of Korea)

Keywords: Copper, Single crystal, Transparent conductive electrode, Flexible electrode

***Supplementary Information***


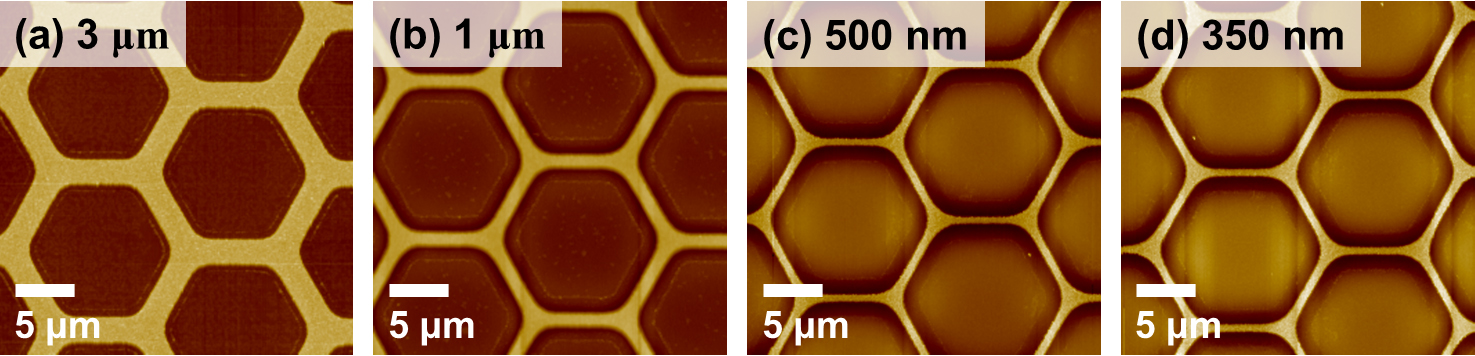


**Figure S1.** AFM topography images of SCu mesh electrodes having a line width of (a) 3 μm, (b) 1 μm, (c) 500 nm, and (d) 350 nm.

Figure S1 shows AFM topography images of SCu mesh electrodes of various line widths. Line widths of 3 μm, 1 μm, 500 nm, and 350 nm were fabricated by varying the etching time of the wet-etching process. All of the SCu honeycomb mesh electrodes exhibited high aspect ratios and well-defined structures.


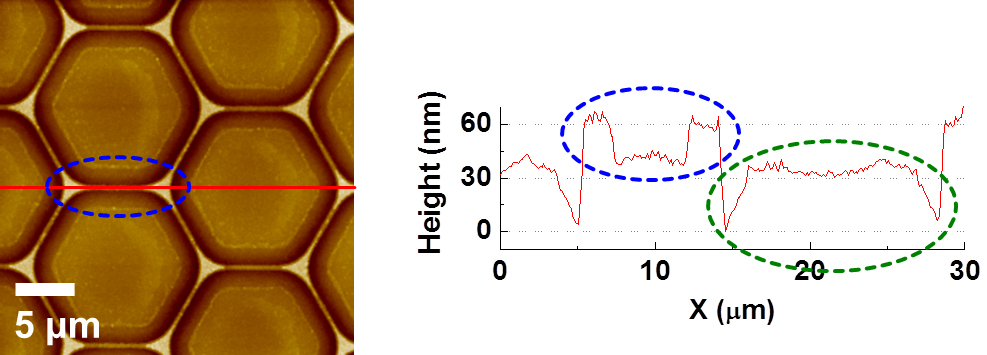


**Figure S2.** AFM topography image and height profile of an SCu mesh electrode with a line width of 200 nm. In the height profile, the collapse of the conductors forming the honeycomb structure is indicated by the dashed blue line.

Figure S2 shows an AFM topography image of an SCu mesh electrode with a line width of 200 nm and a height profile corresponding to the solid red line in the AFM topography image. Similar to the case of Figure S1, Figure S2 shows a well-defined honeycomb structure; however, the conductor path marked by the blue dashed line shows signs of irregularities. The initial height of the conductor path is approximately 60 nm; however, the structure was unable to withstand the undercutting during the etching process and the conductor path collapsed. In the area marked with the dashed green line, the polyimide substrate swelled in the region exposed to the diluted acid solution because of water absorption by the polyimide during the wet-etching process. To flatten the polyimide substrate, we would have to reduce the etching time by increasing the acid concentration or include a drying process after the annealing procedure.


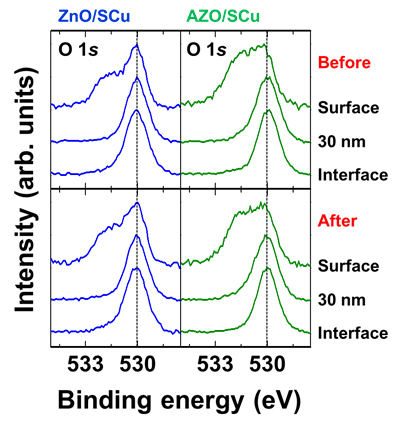


**Figure S3.** XPS O 1*s* depth profiles of in the ZnO and AZO capping layers of the hybrid electrodes before and after the electrodes were thermally annealed at 200 °C. The depth profiles were measured at the surface, at a depth of 30 nm, and at the interface between the capping layer and the SCu mesh .

Figure S3 shows the depth profiles obtained from the XPS O 1*s* spectra of the ZnO and AZO capping layers of the hybrid electrodes. The binding energies were measured before and after the electrodes were thermally annealed at 200 °C in air. In both the ZnO and AZO capping layers, the binding energy corresponding to Zn-O was observed at 530.0 eV, and the peak intensities in the broad spectra related to carbonates (531.0 eV) and hydroxides (531.5 eV) were increased after the electrodes were thermally annealed in air. However, no changes in the XPS spectra or in the binding energies related to carbonates or hydroxides were observed in spectra collected below the surface. According to these results, contamination due to chemical reactions with the external environment was largely prevented by the oxide capping layers and oxidation could not appreciably penetrate the surface of either the ZnO or the AZO capping layer.
